# Oxidation‐resistant and thermostable forms of alpha‐1 antitrypsin from *Escherichia coli* inclusion bodies

**DOI:** 10.1002/2211-5463.12515

**Published:** 2018-09-17

**Authors:** Wei Zhu, Lanfen Li, Mingjing Deng, Bo Wang, Mengfei Li, Guofang Ding, Zuisu Yang, Dan Medynski, Xiaotao Lin, Ying Ouyang, Jirui Lin, Luyuan Li, Xinli Lin

**Affiliations:** ^1^ Key Laboratory for Microorganisms and Biotransformation College of Life Science South‐Central University for Nationalities Wuhan China; ^2^ State Key Laboratory of Protein and Plant Gene Research School of Life Sciences Peking University Beijing China; ^3^ Key Engineering Research Centers of Marine Organisms Medical Products Food and Medicine School of Zhejiang Ocean University Zhoushan China; ^4^ Marine Fisheries Research Institute of Zhejiang Province Zhoushan China; ^5^ Cardinal Intellectual Property LLC Oakland CA USA; ^6^ Shenzhen YHLO Biotech Co., Ltd. Shenzhen China; ^7^ Wuxi Biortus Biosciences Co., Ltd. Jiangyin China; ^8^ School of Software Huazhong University of Science and Technology Wuhan China; ^9^ State Key Laboratory of Medicinal Chemical Biology Nankai University College of Pharmacy Tianjin China

**Keywords:** *Escherichia coli* inclusion body refolding, mutant alpha‐1 antitrypsin, protease inhibitor, protein therapeutic, recombinant protein

## Abstract

Native α1‐antitrypsin (AAT) is a 52‐kDa glycoprotein that acts as an antiprotease and is the physiological inhibitor of neutrophil serine proteases. The main function of AAT is to protect the lung from proteolytic damage induced by inflammation. AAT deficiency (AATD) is a codominant autosomal disorder caused by pathogenic mutations in SERPINA1 gene, leading to reduced levels of serum AAT. The deficiency is known to increase the risk of pulmonary emphysema and chronic obstructive pulmonary disease as a consequence of proteolytic imbalance induced by inflammation, associated in many instances with cigarette smoking and other environmental hazards. Currently, the available therapy for lung disease associated with AATD is serum purified human AAT injected into patients on a weekly basis. It would be advantageous to replace serum‐derived AAT with a recombinant version which is stable and resistant to oxidation. We have expressed AAT in *Escherichia coli* as inclusion bodies and developed a highly efficient refolding and purification process. We engineered a series of mutant forms of AAT to achieve enhance thermostability and oxidation resistance. Moreover, we synthesized an active form of AAT via cysteine‐pegylation to achieve a markedly extended half‐life *in vivo*. The resulting molecule, which retains comparable activity to the wild‐type form, is expected to be an improved therapeutic agent for treating hereditary emphysema. In addition, the molecule may also be used to treat other types of emphysema caused by smoking, cystic fibrosis, pulmonary hypertension, pulmonary fibrosis, and chronic obstructive pulmonary disease.

**Abbreviations**
AAT: alpha‐1 antitrypsin
CatG: cathepsin G
COPD: chronic obstructive pulmonary disease
HLE: human leukocyte elastase
mutein: mutant protein
PPE: porcine pancreatic elastase
Pr3: proteinase 3

Alpha‐1 antitrypsin (AAT) was discovered as early as 1963 to be linked to hereditary emphysema 1. In the 1970s, researchers discovered the function of AAT and established AAT deficiency (AATD) as a risk factor for emphysema 2, 3. In the past decades, the molecular pathology of the cirrhosis and emphysema associated with AATD has been extensively studied 4, with better understanding of the genetic disorder as contributing factors to the development of chronic obstructive pulmonary disease (COPD), bronchiectasis, liver cirrhosis, and panniculitis 5.

Previous research has established that human leukocyte elastase (HLE) is a serine protease released from the azurophilic granules of the neutrophil as part of the normal inflammatory response 6. Under normal homeostatic conditions, AAT serves as an important regulator of proteolysis by HLE, thereby preventing damage of the lung alveolar matrix. AAT is a 52 kDa glycoprotein synthesized primarily in the liver, but also in neutrophils, monocytes, and macrophages. AAT is secreted into the blood plasma, but its primary site of action is in the lung parenchyma 7. Besides HLE, AAT also inhibits two other proteases released into the lungs by neutrophils, namely cathepsin G (CatG) and proteinase 3 (Pr3). CatG and Pr3 may also contribute to lung damage by breaking down elastin and other extracellular matrix proteins. By inhibiting the function of these two proteases, AAT may in turn prevent this potential damage. That being said, HLE is considered to be the enzyme primarily responsible for lung damage 8. The normal biological function of AAT is essential for human health and has been called ‘a guardian of vascular tissue’ 9.

In the lung, neutrophils release a wide range of defensive molecules as part of an inflammatory response, including reactive molecular oxygen species, cationic peptides, eicosanoids, and proteolytic enzymes 10. Paradoxically, unregulated release of these molecules can lead to serious lung damage, leading to a protease–antiprotease hypothesis 5. During acute‐phase inflammatory response, AAT rises more than fourfold to neutralized released proteolytic enzymes to prevent further tissue damage. However, the activity of AAT is also needed to be regulated to keep the protease–antiprotease balance in check 11. One of the function of the reactive molecular oxygen species secreted by neutrophils may be to neutrolize nearby AAT for biological regulation 12. And in turn, AAT may be designed to be sensitive to oxidation in order to prevent ‘overkill’ important proteolytic activities.

It is estimated that there is a total of 253 404 AAT pathogenic deficient mutation patients (Pi*ZZ) worldwide 13: 119 594 in Europe, 91 490 in America and the Caribbean, 3824 in Africa, 32 154 in Asia, 4126 in Australia, and 2216 in New Zealand. These patients suffer from hereditary emphysema and may be treated with the AAT augmentation therapy 14. Globally, it is estimated that about 3 million deaths were caused by chronic obstructive pulmonary disease (COPD) in 2015, according to the World Health Organization, and in many cases can be treated with AAT therapy. However, the AAT drugs which are currently on the market (Prolastin^®^, Aralast^®^, Zemaira^®^, Glassia^®^) are all derived from human serum and can satisfy less than 10% of the AAT‐deficient population only 14. Because of the limited supply of human serum and therefore native AAT, it has not been adequately tested for its beneficial effects in other respiratory disorders, which include emphysema caused by smoking, cystic fibrosis, pulmonary hypertension, pulmonary fibrosis, and COPD 7, 15, 16. Worldwide, it is estimated that there are at least 116 million alpha one carriers (PiMS and PiMZ: M, normal genetic Pi Type; S, lower than normal; Z, lower than S) and 3.4 million with deficiency allele combinations (PiSS, PiSZ, and PiZZ) 14, 17, all of whom are sensitive to environmental conditions to develop COPD.

Many different expression systems have been used to express AAT. These include bacteria 18, 19, yeast 20, plant cultures 21, 22, 23, and transgenic sheep 24, 25, 26, 27. Because of the large dose required for hereditary emphysema (4–6 g·week^−1^ per patient injectable–60 mg·kg^−1^·week^−1^ 28, and up to 250 mg daily for aerosolized lung delivery 29), the essential requirements for manufacturing AAT are scalability—megakilos of pharmaceutical grade drug required per year—and cost control—to make the drug affordable to patients. *Escherichia coli* expression is one of the most cost‐effective production method for recombinant protein drugs. However, AAT has a tendency to form insoluble inclusion bodies when over‐expressed in bacteria, thus limiting the scalability of soluble expression. In this study, using the efficient inclusion refolding technologies developed in our laboratory 30, we refolded and purified recombinant AAT from inclusion bodies. In addition, with the goal of producing an improved therapeutic agent, we constructed several mutant forms of AAT, according to previous research and our own design. Previous research has shown that a single mutant form of F51L improved thermal stability 31, 32, and a double‐mutant M351V/M358V can reduce oxidation‐induced inactivation 12. Accordingly, we designed a triple‐mutant F51L/M351V/M358V, resulting in a more stable, oxidation‐resistant form of AAT which still retains activity comparable to that of wild‐type. We have also produced and purified a pegylated form of AAT for *in vivo* application.

## Results

### Expression constructs and mutant screening

**Figure 1:**
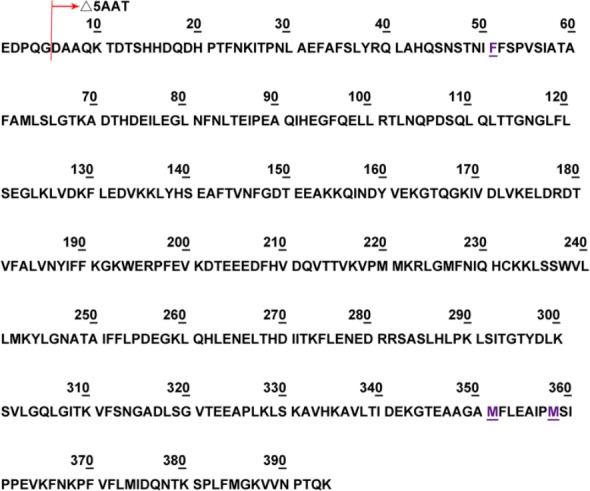
Protein sequence of the mature AAT along with the amino acid substitution positions of the designed muteins. The starting position of Δ5AAT is shown, and in the *Escherichia coli* expression vector, an additional starting Met was installed. Positions of amino acid changes are labeled with underlines. The mutant numbers in the text are followed with the mature, full‐length AAT 42. The nucleotide changes of the three mutants are as follows: F51L, TTT → CTG; M351V/M358V, ATG → GTG/ATG → GTG; and F51L/M351V/M358V, TTT → CTG/ATG → GTG/ATG → GTG.

For *E. coli* expression, we first constructed a full‐length, wild‐type mature form of AAT (Fig. 1) in a pET‐11 (Novagen) vector. This resulted in very low level of expression. We then made N‐terminal deletions according to a published literature 18 and expressed the Δ5AAT (deleting the first five amino acid of the mature AAT) and Δ10AAT (deleting the first 10 amino acid of the mature AAT); both expressed well. The Δ5AAT was chosen for both high level of expression and high percentage of refolding. In order to develop a better therapeutic agent, we constructed three mutants for characterization according to previous research and our own design, as described in the Introduction. The first mutant, F51L 31, 32, results in increased thermostability; the second is a double‐mutant M351V/M358V designed to reduce oxidation‐induced inactivation 12; and the third is a combination mutant with both stabilization and oxidation‐resistant properties (F51L/M351V/M358V). All of the mutants were constructed using standard PCR mutagenesis techniques and verified by sequencing. The starting position of the Δ5AAT protein sequence, along with specific amino acid substitutions, is shown in Fig. 1.

### *Escherichia coli* expression

**Figure 2:**
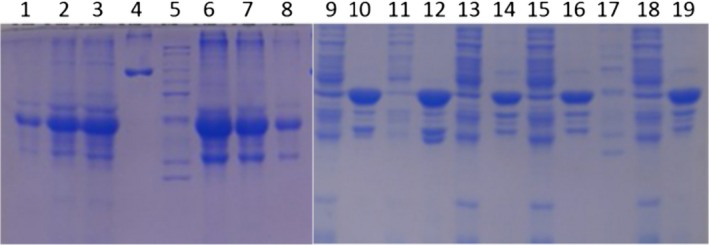
SDS/PAGE of expressed wild‐type, F51L, M351V/M358V, and F51L/M351V/M358V muteins. Proteins were visualized by Coomassie blue staining. Small‐scale expression was performed. Lanes 1–3: purified wild‐type AAT inclusion bodies were loaded 1, 3, 5 μL, respectively. Lane 4: BSA marker. Lane 5: MW standard. Lanes 6–8: Purified F51L mutein inclusion bodies were loaded 5, 3, 1 μL respectively. Lanes 9–19: expression test of soluble and insoluble cell extract. Lanes 9, 11: wild‐type, soluble extract; lanes 10, 12: wild‐type, insoluble extract. Lanes 13, 15: M351V/M358V, soluble extract; lanes 14, 16: M351V/M358V, insoluble extract. Lane 17: MW standard. Lane 18: F51L/M351V/M358V, soluble extract. Lane 19: F51L/M351V/M358V, insoluble extract.

Both wild‐type and mutant ATT were expressed to high levels in the *E. coli* expression host (Fig. 2), which shows that when proper growth media were used, all of the expression constructs can be expressed in high yield, although mostly in the insoluble inclusion body form.

### Refolding, purification, and activity

**Figure 3:**
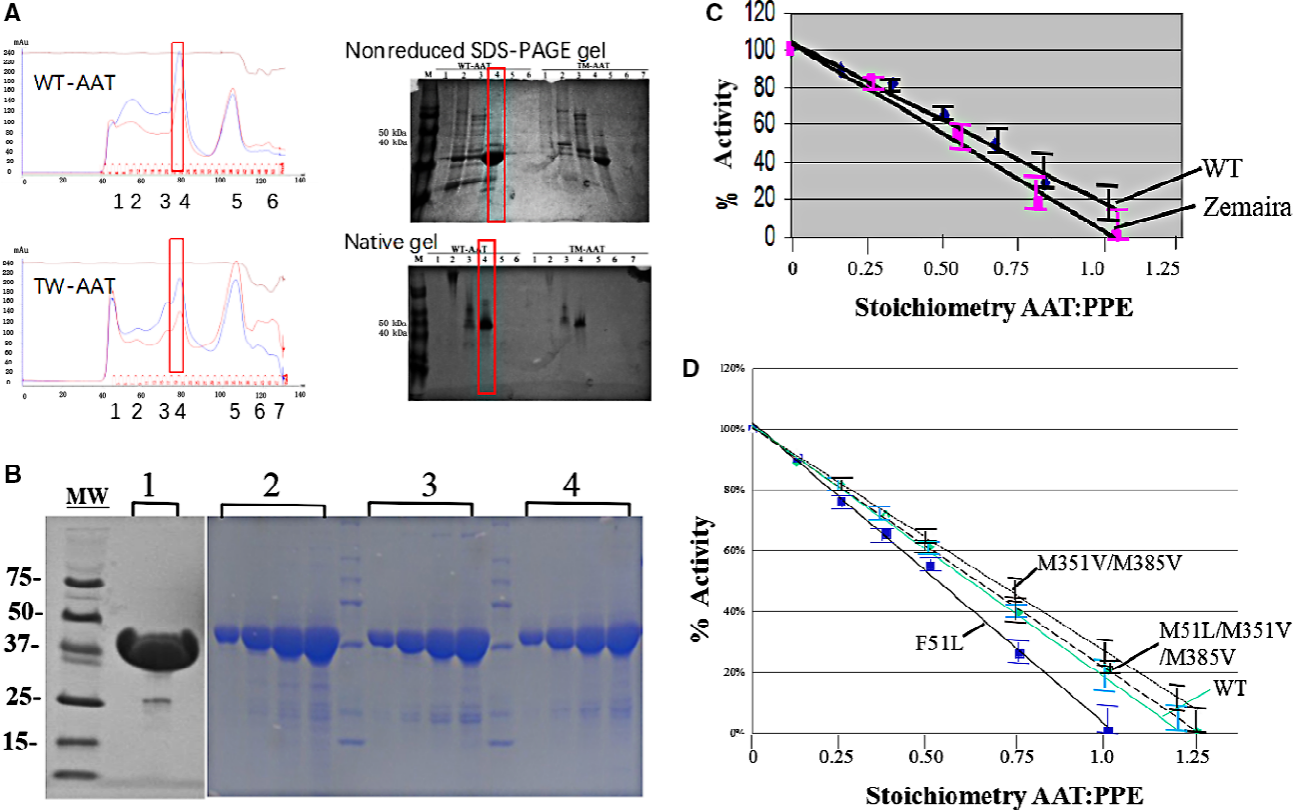
SDS/PAGE of purified recombinant AAT and muteins (A, B), and stoichiometric inhibition of PPE (C, D). (A) Superdex S200 Size Exclusive Column Chromatography (SEC) of refolded wild‐type (WT) and triple‐mutant (TM) AAT. The left represents WT and TM‐AAT SEC column adsorption profiles, and the right is the nonreduced and native gel of some of the corresponding fractions. Peaks 1 and 2 are unfolded, aggregation proteins, and peak 4 is a folded peak, which shows as monomer both in nonreduced and native gels. (B) Purified samples were concentrated and applied to the SDS/PAGE and stained with Coomassie Blue. Lane 1: wild‐type AAT; lanes 2–4: increasing quantities were loaded for purity check (1, 2, 4, 6 μg, respectively); Lane 2: F51L; Lane 3: M351V/M358V; Lane 4: F51L/M351V/M358V. Molecular weight markers (MW) indicated. (C) Activity of recombinant AAT (WT) compared with Zemaira, a commercial native AAT drug as a reference. (D) Activity of purified muteins, as compared to the WT. The data represent SD of the means of triplicate observations.

The purified inclusion bodies can be refolded in various pH and buffers. The general purification procedure includes two steps. The first step involves concentrating the refolded materials by ultrafiltration and then separating the unfolded or partially refolded AAT from folded monomer by SEC column chromatoraphy (Superdex 200 or Sephacryl 300; GE Healthcare, Life Sciences, Shanghai, China). The second step utilizes either ion exchange or hydrophobic interaction column chromatography procedures. Figure 3A shows Superdex 200 purification profile of refolded wild‐type (WT) and triple‐mutant (TW) AAT (left panel) and the nonreduced and native gels of SEC fractions (right panel). The figure clearly shows the monomeric nature of the refolded protein. Figure 3B shows a nonreducing SDS/PAGE of purified AAT SDS/PAGE of purified AAT sample obtained by a hydrophobic interaction chromatography. Note that the highly concentrated protein is mostly monomeric, with a trace of dimeric form in the absence of reducing agent when running SDS/PAGE. The nonreduced SDS/PAGE has been routinely used to distinguish folded and nonfolded proteins in our laboratory 30. The purified recombinant AAT displayed nearly identical inhibitory activity when compared to therapeutically available human AAT (glycosylated Zemaira^®^ from Aventis Behring LLC). The porcine pancreatic elastase (PPE) is inhibited completely at a stoichiometric ratio of AAT : PPE of ~ 1.07 : 1 (Fig. 3C), indicating that the purified recombinant AAT is fully active. The purified mutant proteins (muteins) are shown in Fig. 3D. The activity of the muteins is comparable with that of the wild‐type.

### Cys232 pegylation

**Figure 4:**
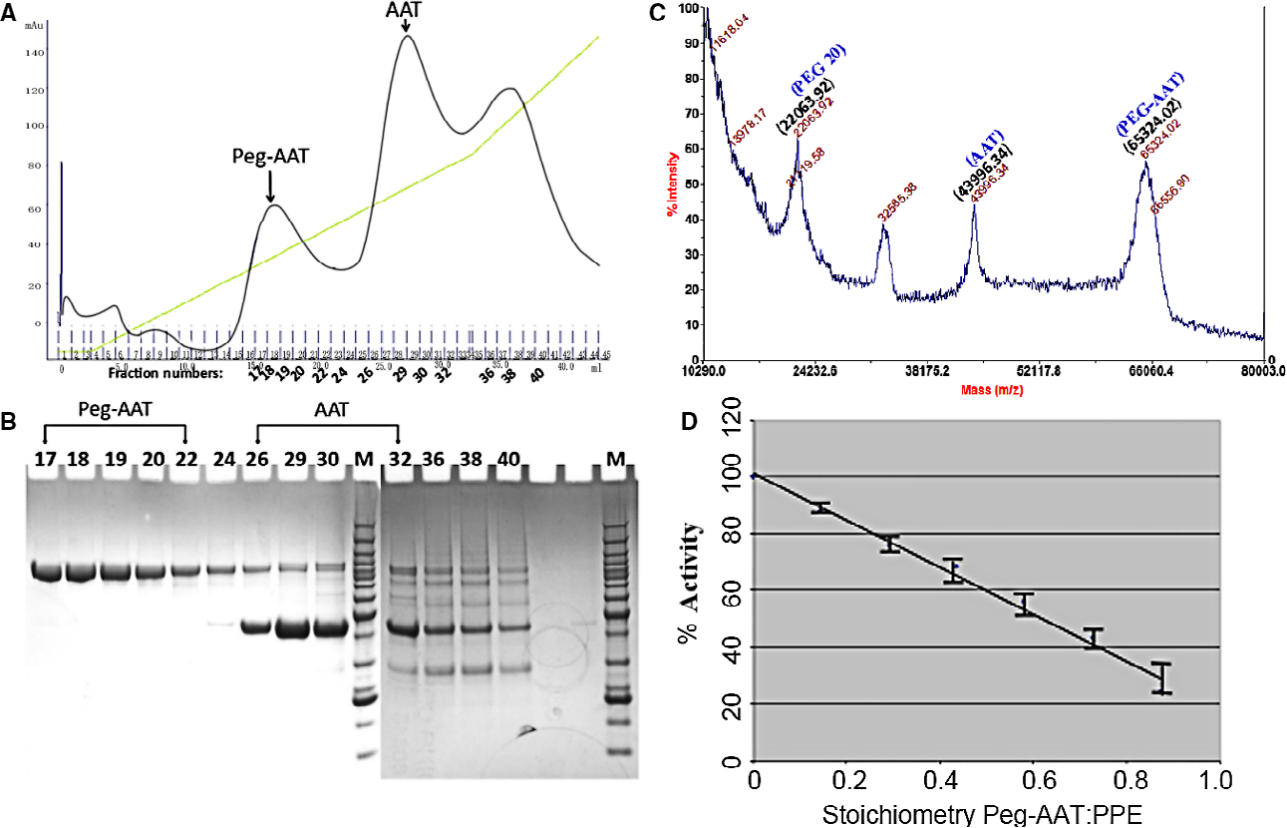
Cys^232^ pegylation, purification, and properties. (A) Cation exchange column profile of pegylated AAT. (B) Nonreduced SDS/PAGE of fractions from the Q XL column. Numbers correspond to gradient fractions shown in (A). (C) MALDI‐TOF mass spectrometry of a sample after pegylation reaction. The molecular weight of each of the indicated peaks is depicted at the top of the respective peak. (D) Purified, pegylated AAT was shown to have normal inhibitory activity in blocking PPE. The data represent the SD of means of triplicate observations.

Purified rAAT was pegylated at the unique Cys232 position according to a published method 33. The efficiency of pegylation was in the range 50–65% across multiple experiments. Molecular models indicate that this unique cysteine is partially exposed to aqueous solvent and is not near the domain of AAT which interacts with elastase (see Fig. 7 in the Discussion section). After pegylation, unreacted maleimide‐PEG (~ 20 kDa; Nektar Therapeutics) and nonpegylated AAT were separated from pegylated AAT by anion exchange chromatography (Q‐HiTrap; Amersham‐Pharmacia, Shanghai, China). Figure 4A shows a Q‐HiTrap purification profile, Fig. 4B shows SDS/PAGE, Fig. 4C shows a MALDI‐TOF mass spectrometry, and Fig. 4D shows the normal functionality in blocking PPE of the pegylated rAAT polypeptide. This experiment demonstrates that pegylated AAT can be separated from unpegylated AAT and free unreacted mPEG20 simply by running the mixture on a salt gradient. The success of the pegylation reaction was demonstrated by SDS/PAGE and MALDI‐TOF mass spectrometry. For instance (Fig. 4C), the molecular weight of the AAT polypeptide (unpegylated) is 43 996.34 daltons, while the molecular weight of the pegylation reagent Mal‐PEG 20 is 22 063.92 daltons. The successfully pegylated rAAT has a molecular weight of 65 324.02 daltons, in close agreement with its predicted molecular weight. This indicates that the rAAT has been successfully pegylated. The ‘free’ rAAT and Mal‐PEG 20 mass is probably generated during the ionization break down of the mass spectrometry process.

### Thermal stability

**Figure 5:**
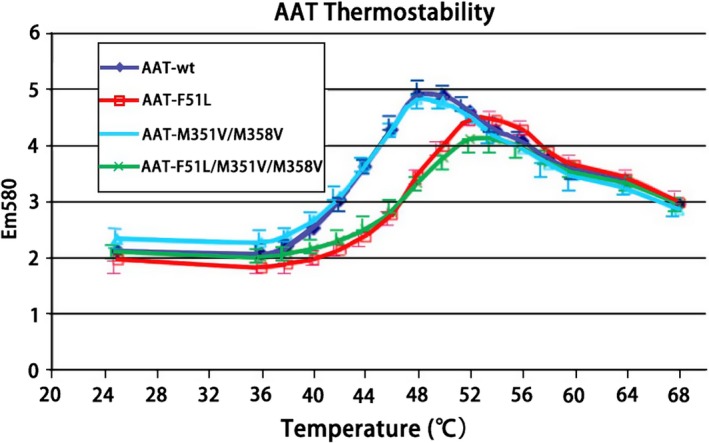
Comparison of thermal stability of wild‐type and mutant AAT. Details of the experiments were described in Materials and methods. The figure shows the counts of fluorescence (*y*‐axis) against the temperature (*x*‐axis, °C). The data represent means of duplicate observations.

In order to increase thermal stability and oxidation resistance, we constructed three kinds of mutants. The first is a F51L single mutant which result in thermal stability; the second is a M351V/M358V double mutant which results in oxidation resistance; and the third is a F51L/M351V/M358V triple mutant which confers both thermal stability and oxidation resistance. To compare the thermal stability of the wild‐type and muteins, we used a fluorescent‐based thermal denaturation assay, as shown in Fig. 5. When the fluorescent dye, in this case SYPRO Orange, binds to a hydrophobic surface, its fluorescence dramatically increases. As the protein denatures with increasing temperature, its hydrophobic surfaces become exposed and bind to the dye, resulting in increased fluorescence. Further increase in temperature separates the dye and the protein, resulting in a denaturation peak. Figure 5 shows that the wild‐type and the M351V/M358V mutein have thermal denaturation at about 48 °C, while both muteins that contain F51L, the single mutant F51L and the triple‐mutant F51L/M351V/M358V, have increased denaturation temperature of about 54 °C. The results show that both single and triple muteins containing F51L greatly increased the thermal stability of AAT.

### Oxidation resistance

**Figure 6:**
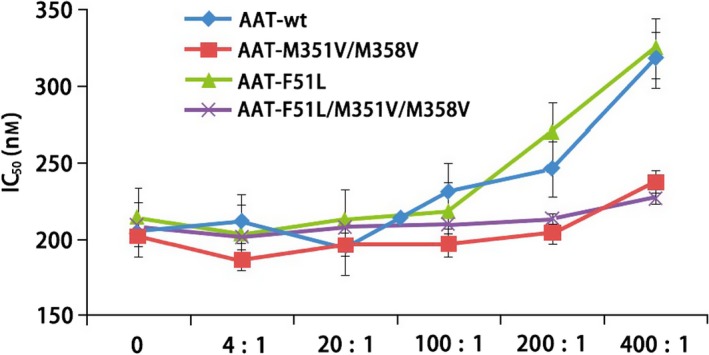
Oxidation‐resistant assay. The generation of aminolytic activity was monitored (at 405 nm) at 37 °C in 10‐s intervals for 20 min using SpectraMax 250 microplate reader (Molecular Devices). The IC_50_ (*y*‐axis) of each H_2_O_2_‐treated AAT or its mutants were determined using grafit version 7 (Erithacus Software, http://www.erithacus.com). The *x*‐axis shows the molar ratio of H_2_O_2_ and AAT (H_2_O_2_ : AAT, from 4 : 1 to 400 : 1). The data represent the SD of means of duplicate observations.

In addition to constructing the oxidation‐resistant mutant M351V/M358V, we also made a combination mutant of F51L/M351V/M358V, which is predicted to be oxidation‐resistant and also have increased thermal stability. As described above (Fig. 5), the triple mutant is more thermal stable, and the oxidation‐resistant experiment indicates that both muteins containing M351V/M358V are more oxidation‐resistant. In Fig. 6, we measured the AAT activity with IC50 value toward inhibition of PPE. When AAT loses specific activity due to oxidation, increased amount was required to inhibit half of the PPE activity, and therefore, the IC50 value increases. The figure shows that when the molecular ratio of H_2_O_2_ to AAT increased from 4 : 1 to 400 : 1, native AAT, and the F51L started to lost its *in vitro* activity of inhibiting PPE, but both muteins containing M351V/M358V are resisting oxidation up to a 400 : 1 ratio. According to the literature, the *in vitro* oxidation‐resistant properties of the mutant protein as well as the increased thermostability can be translated into enhanced *in vivo* stability and therefore may be used as a better drug candidate 34.

## Discussion

The goal of this study was to develop a recombinant AAT therapeutic by using an efficient and cost‐effective expression system to generate rationally designed muteins for both increased thermal stability and oxidation resistance. In addition, we were also able to make chemical modifications to the protein which predicts to result in a longer *in vivo* half‐life. The *E. coli* expression and refolding system we developed can achieve high yields of highly pure protein at a relatively low cost. Despite this, there are significant hurdles for medical application of the recombinant wild‐type protein. The first problem is that by design, AAT is prone to oxidation, and is unstable under physiological conditions, due to its functional requirement 12, 31, 34. In this study, we tried to solve this problem by constructing and selecting mutant proteins that are more stable and oxidation‐resistant, and are therefore more suitable for pharmaceutical development. The second problem is that because the *E. coli* produced AAT is not glycosylated, the *in vivo* half‐life of the unglycosylated AAT is much shorter than the native, glycosylated one. We therefore designed a pegylation procedure for the *E. coli* refolded AAT.

**Figure 7:**
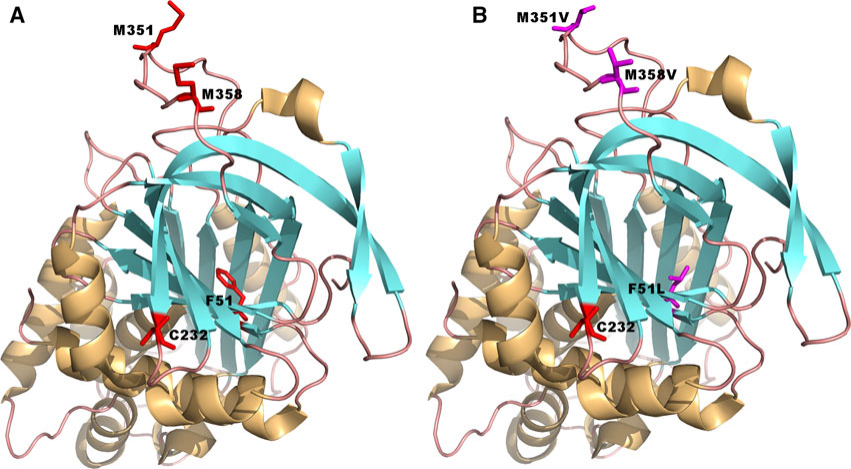
Three‐dimensional structure of wild‐type (A) and triple‐mutant (B) AAT from Lomas *et al*. indicating site of proposed antioxidant mutations (Met^351^ and Met^358^, P8 and P1 of the active site, respectively), stability mutation (Phe^51^) buried deep in the hydrophobic core of the molecule, and Cys pegylation (Cys^232^, exposed on the surface but not obstructing the active site). Modeling software coot http://www2.mrc-lmb.cam.ac.uk/personal/pemsley/coot/ and PDB coordinates http://www.rcsb.org/pdb/search/structidSearch.do?structureId=1QLP were used to formulate this protein model based on solved crystal structure. The whole structures are shown as a cartoon representation. The wild‐type and mutant‐type residues are shown in a stick representation. The picture was generated using the pymol software (http://www.pymol.org).

More specifically, we first expressed and refolded a F51L mutein which resulted in a more stable rAAT. As shown in Fig. 7, the wild‐type phenylalanine residue at position 51 is buried in the hydrophobic core of the molecule, far away from the active site. After a round of nonspecific chemical mutagenesis and selection, Kwon *et al*. 31 demonstrated that aliphatic amino acid substitutions at this position confer dramatically increased thermal stability of AAT without prompting inactivation, aggregation, or a change of association kinetics with the elastase. The nonglycosylated *E. coli*‐expressed mutant was shown to slow down heat inactivation more that 10‐fold at 57 °C such that the mutant behaved like plasma‐derived glycosylated AAT. In this study, we replaced this residue with a leucine (F51L) after modeling with a crystal structure (Fig. 7). Results show that this substitution confers dramatically increased thermal stability of AAT (Fig. 5).

Although some genetic variants in located at adjacent positions, such as Phe^51^‐Phe^52^ 35 or Ser^53^‐Phe 36 deletions can cause *in vivo* aggregation of native AAT, the F51L mutein, as well as the triple mutant in this study, has no aggregation issues in all of our experiments.

The second kind of mutation results in oxidation resistance. It is well known that AAT is very sensitive to oxidation due to its *in vivo* functional regulation requirements 12, 37. Oxidation is known to occur from the components inhaled in cigarette smoke such as hydrogen peroxide, etc., and it has been hypothesized that reduction in functional AAT in the lungs of smokers contributes to lung pathophysiology. The most susceptible residues are Met351 and Met358 12, 37, which happen to be the P8 and P1 positions of the AAT binding site (Fig. 7), respectively. Conservative substitution of each of these positions with another aliphatic amino acid such as valine 12 resulted in a molecule that was markedly resistant to oxidation by hydrogen peroxide and did not significantly affect AAT's association kinetics or binding to target enzyme neutrophil elastase 12, 34. Our results also show that a double mutant, M351V/M358V, confers a marked increase in oxidation resistance (Fig. 6).

In addition, we have constructed a novel combination of stabilization and oxidation‐resistant mutant, F51L/M351V/M358V. After refolding, the triple mutant is fully active and shows both enhanced thermostability and resistance to oxidation (Figs 5 and 6).

The *in vivo* half‐life of the *E. coli* produced, unglycosylated rAAT is much shorter than native, glycosylated AAT. For instance, the plasma half‐life of glycosylated rat AAT measured in rat serum is 170 min, while it is only 30 min for the unglycosylated form of this molecule 38. One of the most effective methods for extending the *in vivo* half‐life and to reduce immune reaction of a protein is polyethylene glycol conjugation (pegylation) 39. Pegylation is a proven method for extending *in vivo* half‐life and reducing immunogenicity and has been successfully used in biological drug developments for decades 40. As shown in Fig. 4, we have successfully pegylated Cys232 of the wild‐type rAAT according to the published procedure 33, 41. Cys232 is the only cysteine in AAT, which exists as a monomeric molecule (Fig. 7), and is therefore accessible to a site‐specific pegylation. The pegylated material behaved like the wild‐type AAT in inhibiting PPE (Fig. 4) *in vitro*, with similar association rates.

In summary, we have expressed recombinant AAT in *E. coli* with high yields in inclusion body forms, purified and refolded the inclusion bodies, and developed highly efficient refolding and purification procedures for pharmaceutical drug development. In addition, we produced a novel triple‐mutant form of AAT with enhanced thermal stability and oxidation resistance, and also a pegylated form to further extend the *in vivo* half‐life. The resulting entity could potentially have superior druggable properties for a range of medical applications than the native AAT from human serum.

## Materials and methods

### Cloning

Both of the full‐length, wild‐type mature forms, the Δ5AAT and Δ10AAT, and the mutants were cloned into a pET‐11 (Novagen, http://www.novagen.com) vector with standard molecular biology techniques.

### Expression

For wild‐type and F51L mutein expression, the *E. coli* expression clone was initially expanded and then inoculated into 1.0 L of LB media containing tryptone 10 g, Yeast extract 5 g, NaCl 10 g, with 50 mg ampicillin, induced with 0.5 mm IPTG at OD_600_ = 0.6, and allowed to express for 3 h at 37 °C.

For M351V/M358V and F51L/M351V/M358V muteins, the *E. coli* expression clones were initially expanded in LB media and then inoculated into 1.0 L of TB media containing tryptone 12 g, Yeast extract 24 g, glycerol 4 mL, 17 mm of KH_2_PO_4_ plus 72 mm of KH_2_PO_4_, with 50 mg ampicillin, induced with 0.5 mm IPTG at OD_600_ = 0.6, and allowed to express for 4 h at 37 °C.

At the end of the growth, the broth was spun down and thoroughly resuspended in a lysis buffer containing 20 mm Tris, 50 mm NaCl, 2.5% glycerol, pH8.0. Lysozyme (0.3 mg·mL^−1^) was then added and the resulting suspension was stored at −80 °C for inclusion body purification.

### Inclusion body purification

The frozen inclusion bodies were thawed and bacteria were thoroughly broken by ultrasonic disruption. The inclusion bodies were purified by washing five times with a TN buffer (100 mm Tris, 250 mm NaCl, pH 8.0) containing 1% Triton X‐100. The purified inclusion bodies were dissolved in a 8 m urea buffer solution (8 m urea, 0.1 m Tris, 1 mm glycine, 1 mm EDTA, 100 mm β‐mercaptoethanol, pH 10) and stirred gently for about 16 h at 4 °C. The solubilized material was then centrifuged to remove insoluble debris. The purified inclusion bodies were adjusted to a final *A*_280_ = 2.0 using the same 8 m urea buffer as a diluent.

### Refolding

The purified inclusion body solution (I.B.) was rapidly diluted into 20 volumes of a refolding buffer consisting of 20 mm Tris, 10% glycerol, pH 9.0, with a final *A*_280_ = 0.1. The solutions were kept at 20 °C for 16 h and then kept at 4 °C for 2 days before proceeding to purification.

As an example, a detailed procedure for a 4‐L refolding follows.

1. Calculate the number of 4 L refolding buffers to set up by taking the Total OD_280_ of I.B. and dividing by final volume of refolding buffer to an OD_280_ of 0.1.
2. Prepare appropriate number of 4 L buffer: 10% (400 g) glycerol in 20 mm Tris/HCl, filter through a 0.45 filter, adjust to pH 9.0.
3. Prepare the I.B. solution by bringing up the volume with 8 m urea. Each 4 L refolding buffer will need 200 mL of the I.B. solution + 140 μL β‐mercaptoethanol + 300 mg DTT + 60 mg Reduced Glutathione.
4. Rapidly stir the 4 L refolding buffer with a large magnetic stirring bar and add the 200 mL I.B. solution into the buffer gradually so that it is rapidly and completely mixed.
5. Let it sit at room temperature for 2–3 h and store at 4 °C for 2 days.

### Purification

Refolded AAT was concentrated to an *A*_280_ > 20.0 using a tangential flow filtration system and loaded onto a 50/100 (50 mm diameter × 100 cm length) of Superdex 200 (S200) column pre‐equilibrated with a buffer containing 20 mm Tris, 0.15 m NaCl, 0.4 m Urea, 1 mm DTT, 10% glycerol, pH 7.6. The active peak fractions (monomer peak calibrated by molecular weight markers) were pooled and dialyzed against a buffer containing: 20 mm Tris, 5% glycerol, 3 m NaCl, 0.001% Tween 20, 1 mm DTT, pH 7.6. The dialyzed material was loaded into a phenyl sepharose column (a 25 mL column for a 4 L refolded material) equilibrated with the dialysis buffer. The run‐through fractions containing the purified material were concentrated and dialyzed against a buffer containing 20 mm Tris, 5% glycerol, 0.001% Tween 20, 1 mm DTT pH 7.6 to remove the salt (NaCl) in the phenyl buffer. The purified sample was concentrated by ultrafiltration and loaded onto a S200 column again for a final purification. The concentration of the purified material was determined by molar extinction in 6 m guanidine, 20 mm NaPi, pH = 6.5 using a computed extinction coefficient ε280 = 19 060 m ^−1^·cm^−1^ for this specific protein.

Example: the final yield and purity of the S200 and phenyl sepharose purification steps:

**Table:** 

Steps	OD280	Column (mL)	Total OD280	Yield
S200	0.82	451	369	92%
Phenyl	0.57	430	245	61%
Purified	8.3	23	191	48%

### Pegylation

Highly purified AAT was exchanged over a PD‐10 (Bio‐Rad, http://www.bio-rad.com) column pre‐equilibrated with 50 mm NaPi pH = 7.5, 200 mm NaCl to remove DTT and change the pH to 7.5 according to the manufacturer's protocol. The buffer exchange process was usually performed twice to be absolutely certain there were no trace levels of DTT present because this reducing agent will interfere with the pegylation reaction. The buffer‐exchanged AAT was quantitated by molar extinction. Solid PEG‐mal20 (polyethylene glycol maleimide 20; Nektar, Huntsville, AL, USA) stored at –20 °C under argon gas was added to the solution of AAT at a molar ratio of 5 : 1 to 10 : 1 and incubated at 37 °C for 30 min. The reaction was stopped by adding 20 mm DTT and incubating for an additional 5 min at 37 °C. Pegylated AAT (Peg‐AAT) was then dialyzed into 20 mm Tris 8.0, 50 mm NaCl, 1 mm DTT to remove excess salt and then loaded on a 5 mL Q XL HiTrap column. The column was run against a gradient of 0–1000 mm NaCl.

### Activity

Biological activity of AAT produced from the properly folded rAAT wild‐type and muteins was measured by inhibition of HLE or PPE activity *in vitro* using chromogenic substrates. The inhibitory properties were tested and compared to commercially available glycosylated full‐length AAT isolated from human plasma and purchased from Calbiochem (San Diego, CA, USA; Cat. #17825) or Zemaria^®^, manufactured and marketed by Aventis Behring LLC (http://www.aventisbehring.com). The PPE isolated from hog pancreas was purchased from Sigma‐Aldrich (St. Louis, MO, USA; cat. # E7885); and the HLE isolated from human sputum was purchased from Molecular Innovations (Southfield, MI, USA; Cat # HNE). A range of concentrations 0.3–14 nm of AAT is incubated with a fixed concentration 1.4 nm of either HLE or PPE for 15 min at 37 °C, and then, aliquots of the incubate were mixed with 1 mm of the elastase substrates *N*‐succinyl‐ala‐ala‐ala‐*p*‐nitroanilide (chromogenic substrate for PPE; Sigma) or *N*‐methoxy‐succinyl‐ala‐ala‐pro‐val‐*p*‐nitroanilide (chromogenic substrate for HLE; Sigma). The kinetics of hydrolysis of the substrate was monitored at 21 °C at 405 nm using a Molecular Devices Spectrophotometer (Spectramax Plus, http://www.moleculardevices.com.cn). The initial velocity of each reaction was determined, and the percentage activity relative to a control (no AAT or AAT polypeptides) was determined. The percent elastase activity was plotted against the stoichiometric molar ratio of concentrations of AAT polypeptide:elastase used in the corresponding reaction. The precise concentrations of stocks of each form of AAT polypeptides, PPE, and HLE used in the experiment were determined prior to the reactions using each respective polypeptide or protein's known extinction coefficient as obtained using the computer software program protparam from the ExPASY proteomics server at the Swiss Institute of Bioinformatics (http://www.expasy.ch).

Detailed experimental procedures for Fig. 3 are described below. A fixed concentration of PPE (80 μg·mL^−1^) was incubated with a range of concentrations of AAT in separate Eppendorf tubes at 37 °C in 50 mm Tris pH 8.8, 38 mm NaCl, 0.01%Tween 20 for 15 min. Ten microlitre aliquots were pipetted in quadruplicate into a microtiter plate, and then, a multipipetter was utilized to pipette 100 μL aliquots of 1 mm chromogenic substrate *p*‐ala‐ala‐pro‐val‐pNA in the same buffer into the microplate wells. The kinetics of elastase cleavage of the substrate was monitored at 405 nm at 21 °C. The velocity was compared to a control (elastase only) and plotted as % control elastase activity (*y*‐axis) vs stoichiometry of AAT : PPE (*x*‐axis). The concentration of PPE used in the assay was derived from the measurement of extinction coefficient for pure PPE in 6 m guanidine, 50 mm NaPi, pH 6.5 using a Swiss Bioinformatics Institute protparam algorithm (www. expasy.ch). The concentration of AAT was determined by first precisely titrating the concentration of trypsin active sites in a stock solution of trypsin (Sigma) using the fluorescent ‘almost irreversible’ substrate MUGB (4‐methylumbelliferyl‐4‐guanidobenzoate hydrochloride, Fluka) from Novagen. Subsequently, the functional concentration of either of the AAT stocks in blocking the functional sites of trypsin was determined in a stoichiometric assay using the chromogenic substrate BAPNA (*N*‐Benzoyl l‐arg‐4 nitroanilide hydrochloride; Sigma) at 21 °C and 405 nm. It was determined that the functional concentration of either form of the AAT was nearly identical to that determined simply by measuring structurally using the extinction coefficient using the protparam computer algorithm from the Swiss Institute of Bioinformatics website (http://www.expasy.ch), indicating near 100% activity of the purified recombinant AAT.

### Thermal stability

The thermal stability assays were set up in 96‐well plates. Each 110 μL of assay buffer contains 1× PBS buffer, 10% v/v glycerol, 10% DMSO, 5 mm DTT, 50× SYPRO Orange (diluted from 5000× in DMSO; Thermo Fisher Scientific, http://www.thermofisher.com), and 15 μm each of the purified AAT or its muteins. The plates were incubated at 25 °C for 30 min in an incubator, and then, the temperature was elevated in 0.5 °C steps until 70 °C. The florescence was measured at Ex 490 nm, Em 580 nm for 200 ms in each temperature steps. The counts of the florescence were plot against the temperature.

### Oxidation resistance

To test the antioxidation of the AAT and its mutants, 50 μm of each purified AAT or muteins was incubated in PBS buffer with 0, 2, 10, 50, 100, and 200 mm of H_2_O_2_ at 25 °C for 15 min. After incubation, the extra H_2_O_2_ was reduced by adding equal amount of DTT. The treated AAT and muteins were set up for inhibition assay using PPE.

## Author contributions

WZ, ML, DM, XL, and YO performed the bioengineering, biochemical, and biophysical experiments. LL, MD, BW, GD, ZY, JL, and LL performed the kinetic, structural, and other analysis. XL and LL directed the study and wrote the article.

## Conflict of interests

LL and XL may have conflict of interest for future drug development and commercialization activities.
